# Body fat percentage and hypertension risk: A population-based observational and Mendelian randomization study

**DOI:** 10.1097/MD.0000000000048262

**Published:** 2026-04-10

**Authors:** Yinuo Qu, Chunqi Jiang, Muning Wang, Yirui Chen, Zhiyuan Liu, Ziyi Zhang, Hongyu Li, Hongxin Gui, Zixin Zhang, Mengyang Wang

**Affiliations:** a Institute of Pharmacy, Shandong University of Traditional Chinese Medicine, Jinan, China; b Department of Geriatrics, Affiliated Hospital of Shandong University of Traditional Chinese Medicine, Lixia District, Jinan, Shandong, China; c College of Chinese Medicine, Tianjin University of Traditional Chinese Medicine, Jinghai District, Tianjin, China; d School of Public Health and Health Science, Tianjin University of Traditional Chinese Medicine, Jinghai District, Tianjin, China.

**Keywords:** body fat percentage, causal inference, hypertension, Mendelian randomization, NHANES, sex differences

## Abstract

This study aimed to investigate the association and potential causal relationship between body fat percentage (BFP) and hypertension, with a focus on sex-specific thresholds and clinical implications for prevention. We integrated data from the National Health and Nutrition Examination Survey (1999–2018; n = 25,957) and summary-level genome-wide association studies (GWAS) from United Kingdom Biobank and Finnish Genetic Epidemiology Consortium. Multivariate logistic regression, restricted cubic spline, and threshold effect analyses were used to evaluate the nonlinear association between BFP and hypertension in the observational cohort. Two-sample Mendelian randomization (MR) employed single-nucleotide polymorphisms as instrumental variables, with inverse-variance weighted as the primary method; sensitivity analyses (MR-Egger, leave-one-out, heterogeneity tests) assessed robustness. Hypertension prevalence increased progressively from adipopenia (6.5%) to obesity (43.1%). Restricted cubic spline revealed a nonlinear positive association between BFP and hypertension in both sexes (*P* < .001 for nonlinearity). Sex-specific inflection points were identified: 31.32% in males and 41.82% in females. Below these thresholds, each 1% increase in BFP conferred higher odds of hypertension (males: odds ratio [OR] = 1.10; females: OR = 1.16); the effect attenuated above the thresholds. MR analyses confirmed a causal effect of genetically predicted BFP on hypertension (United Kingdom Biobank: OR = 1.003, 95% confidence interval: 1.001–1.005, *P* < .001; Finnish Genetic Epidemiology Consortium: OR = 1.846, 95% confidence interval: 1.587–2.146, *P* < .001), with no evidence of heterogeneity or horizontal pleiotropy. Higher BFP is robustly associated with and likely causally contributes to increased hypertension risk, with distinct sex-specific thresholds. These findings support incorporating BFP (beyond body mass index) into hypertension risk assessment and personalized prevention strategies.

## 1. Introduction

Hypertension is a leading contributor to cardiovascular morbidity and mortality worldwide and remains highly prevalent.^[1]^ Excess adiposity is a major and potentially modifiable risk factor for elevated blood pressure. Body mass index (BMI) is widely used but is an imperfect proxy for adiposity because it cannot distinguish fat from lean mass or capture fat distribution.^[2,3]^ Body fat percentage (BFP) more directly reflects overall adiposity and may better identify individuals with high adiposity despite a normal BMI.^[4]^

Accumulating evidence suggests that BFP is not merely correlated with but mechanistically implicated in blood pressure dysregulation.^[5,6]^ Excess adiposity may increase blood pressure through inflammation, insulin resistance, endothelial dysfunction, and activation of the renin–angiotensin–aldosterone system (RAAS).^[7–9]^ Observational studies across diverse populations have reported positive associations between BFP and hypertension, including among individuals with normal BMI.^[10–13]^ However, residual confounding, reverse causality, and measurement heterogeneity limit causal interpretation.^[14,15]^

Mendelian randomization (MR) can strengthen causal inference by using genetic variants as instrumental variables for BFP, thereby reducing confounding and reverse causality.^[10,11,16]^ MR estimates across cohorts may differ in magnitude due to differences in ancestry, phenotype definitions, and trait scaling.^[17]^

To address these gaps, we triangulated evidence from 2 complementary approaches. First, using NHANES 1999 to 2018, we evaluated the association between BFP and hypertension in a nationally representative sample, accounted for the complex survey design, and assessed potential nonlinearity and sex-specific inflection points.^[12,16,17]^ Second, we conducted two-sample MR using summary statistics from large cohorts (including United Kingdom Biobank [UK Biobank] and Finnish Genetic Epidemiology Consortium [FinnGen]) to test whether genetically predicted BFP is causally associated with hypertension. We hypothesized that higher BFP is associated with higher hypertension risk and that genetically elevated BFP has a causal effect; any inflection points should be interpreted as population-level estimates rather than diagnostic cutoffs, and sex differences should be viewed as descriptive and hypothesis-generating.^[18–20]^

## 2. Materials and methods

### 2.1. Overall study design

**Figure 1.**
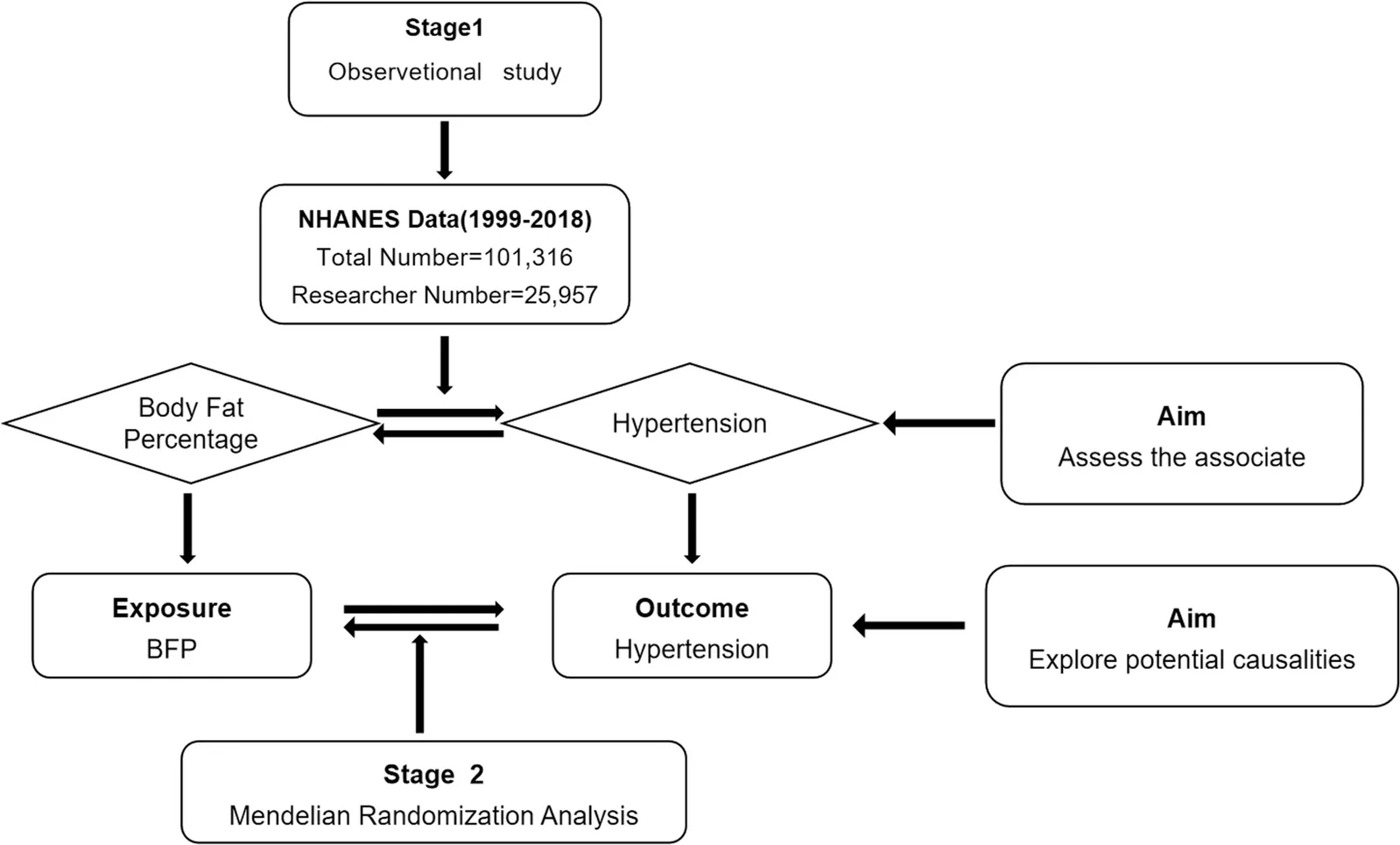
Overall research design flow chart.

This study integrated population-based observational analyses with MR to examine the association between BFP and hypertension (Fig. 1). First, cross-sectional analyses were conducted using data from the NHANES 1999 to 2018 to evaluate the shape, magnitude, and potential nonlinearity of the BFP–hypertension association in a nationally representative U.S. population.^[21–23]^ Second, two-sample MR analyses were performed using summary statistics from large genome-wide association studies (GWAS) to assess the potential causal effect of genetically predicted BFP on hypertension risk.^[24]^

### 2.2. Observational study of NHANES

#### 2.2.1. Data source and study population

The NHANES, conducted biennially by the National Center for Health Statistics of the Centers for Disease Control and Prevention, is a nationally representative, cross-sectional survey designed to assess the health and nutritional status of the noninstitutionalized U.S. civilian population. The survey combines standardized interviews, physical examinations, and laboratory measurements, and employs a complex, multistage probability sampling design to ensure generalizability.^[25]^ All NHANES protocols are reviewed and approved by the National Center for Health Statistics Research Ethics Review Board; because data are publicly available and de-identified, this secondary analysis was exempt from additional institutional review board approval and informed consent requirements.^[26]^

**Figure 2.**
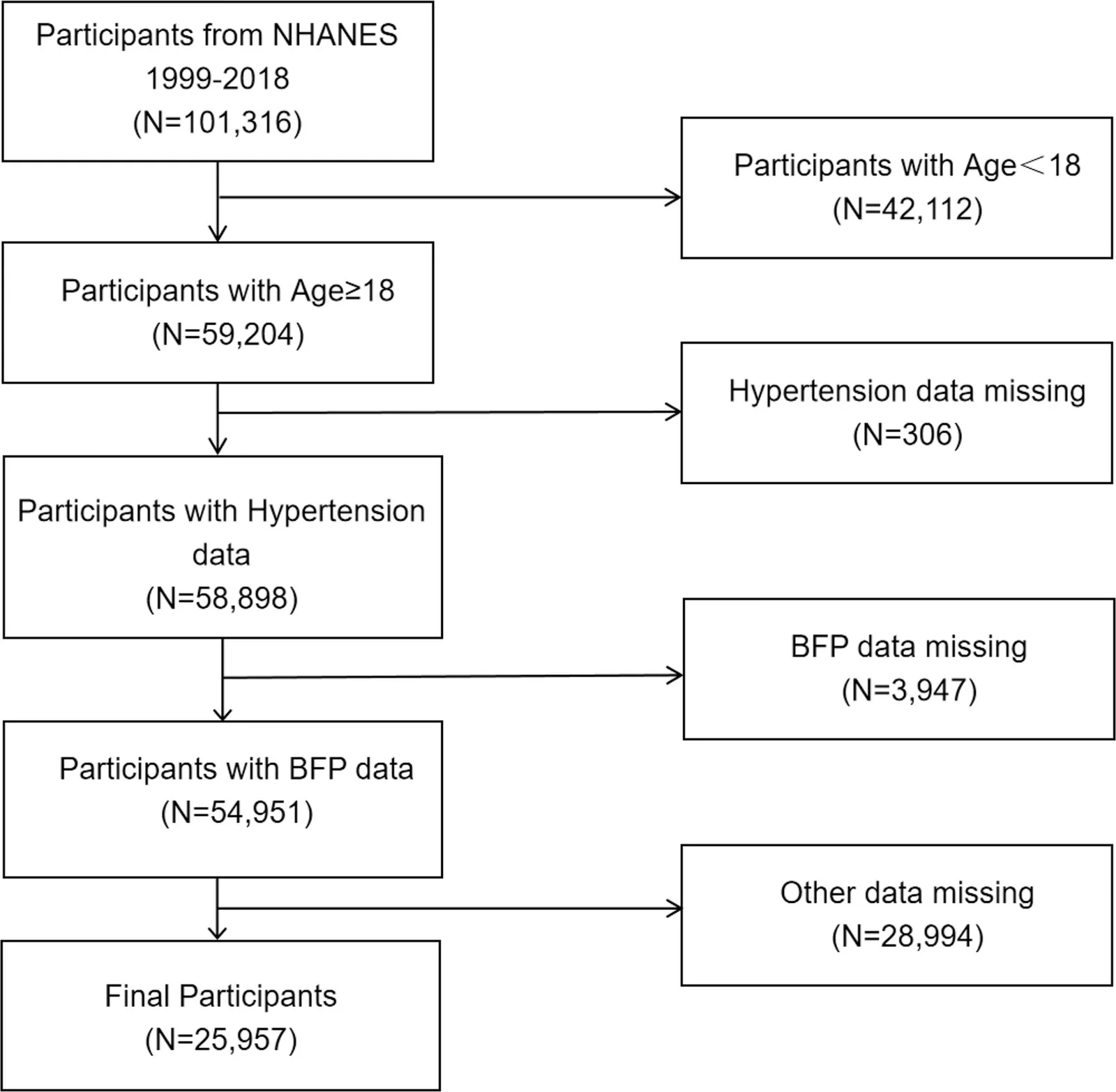
Flowchart of participant selection process.

We pooled data from the 1999 to 2018 NHANES cycles (n = 101,316). Participants were excluded if they were under 18 years of age (n = 42,112), had missing hypertension status (n = 306), lacked BFP measurements (n = 3947), or had incomplete covariate information (n = 28,994). The final analytic sample comprised 25,957 adults with complete data, as detailed in the participant flowchart (Fig. 2). We conducted a complete-case analysis and did not impute missing values. Participants with missing data on BFP, hypertension status, or covariates were excluded from the analytic sample.

#### 2.2.2. Body fat percentage: definition and assessment

BFP was assessed using bioelectrical impedance analysis during the NHANES mobile examination center visit. Bioelectrical impedance analysis estimates total body fat by measuring the resistance of low-intensity electrical current as it traverses lean and adipose tissues, incorporating sex-specific prediction equations that account for height, weight, and body geometry. BFP is calculated as (fat mass/total body mass) × 100%, providing a direct quantification of adiposity that transcends the limitations of BMI: notably its inability to distinguish fat from lean mass or reflect regional fat distribution.^[27]^

For clinical interpretability, we categorized participants into 3 mutually exclusive groups based on sex-specific BFP thresholds: adipopenia: ≤17% in men, ≤30% in women (defined as one standard deviation below the sex-specific sample mean); normal body fat: between adipopenia and obesity thresholds; obesity: >30% in men, >40% in women, consistent with established clinical cutoffs. All BFP data derive from the NHANES Laboratory Files; methodological details are publicly available at https://www.cdc.gov/nchs/nhanes/.

#### 2.2.3. Hypertension: definition and ascertainment

Hypertension was defined per the 2017 American College of Cardiology/American Heart Association guidelines as: systolic blood pressure ≥130 mm Hg or diastolic blood pressure ≥80 mm Hg, based on the average of 3 seated, resting measurements obtained using standardized mercury or aneroid sphygmomanometers; or self-reported physician diagnosis of hypertension (“Has a doctor or other health professional ever told you that you have hypertension?”). This dual-criteria approach enhances case ascertainment while preserving clinical relevance.

#### 2.2.4. Covariate assessment

A comprehensive array of covariates was extracted from NHANES demographic, dietary, and laboratory modules. Demographic variables included sex, age (years), race/ethnicity (non-Hispanic White, non-Hispanic Black, Mexican American, Other Hispanic, and Other race), educational attainment (below high school, high school graduate, and above high school), marital status (married/cohabiting, widowed, divorced/separated, and never married), and poverty-income ratio (<1.3, 1.3–3.5, and >3.5).

Lifestyle factors encompassed smoking status (never, former, and current) and alcohol consumption (none, moderate [<5 drinks/day], excessive [≥5 drinks/day]). BMI was calculated as weight (kg)/height (m^2^) and classified as normal (<25), overweight (25–29.9), or obese (≥30).^[28]^ Comorbidities: diabetes, asthma, coronary heart disease, heart failure, stroke, and cancer, were ascertained via self-report.^[29]^

Laboratory measures, obtained after an overnight fast where applicable, included: hematologic indices: white blood cell count, red blood cell count, neutrophil and lymphocyte percentages, and platelet count; metabolic markers: fasting glucose, triglycerides, sodium (Na^+^), potassium (K^+^), and chloride (Cl^−^); hepatorenal function: albumin, serum creatinine, blood urea nitrogen, aspartate aminotransferase, alanine aminotransferase, and lactate dehydrogenase.^[30–32]^

#### 2.2.5. Statistical analysis

All NHANES analyses accounted for the complex, multistage probability sampling design by incorporating sampling weights, strata, and primary sampling units. Because BFP was measured during the mobile examination center (MEC) component, MEC examination weights were applied. To combine multiple survey cycles from 1999 to 2018, 2-year MEC weights were divided by the total number of cycles and recalibrated according to NHANES analytic guidelines. Continuous variables are presented as weighted means ± standard deviations; categorical variables as weighted frequencies and percentages.

Weighted logistic regression models were used to estimate the association between BFP and hypertension. Restricted cubic spline functions were fitted to assess potential nonlinear relationships, with knots placed at prespecified percentiles of the BFP distribution. Nonlinearity was evaluated by comparing models with and without spline terms using likelihood ratio tests. When evidence of nonlinearity was observed, segmented regression models were applied to estimate inflection points and changes in slope. All analyses were conducted using survey procedures to account for NHANES sampling weights and design. Statistical significance was set at two-sided *P* < .05. All analyses were performed in R using the survey, splines, and EmpowerStats packages.

### 2.3. Mendelian randomization study

#### 2.3.1. Data sources

We conducted two-sample MR analyses using publicly available summary-level GWAS data. Genetic instruments for BFP were derived from a European-ancestry GWAS meta-analysis of 401,772 individuals (GWAS ID: ebi-a-GCST90013975; PMID: 34594039), accessed via the European Bioinformatics Institute and the integrative epidemiology unit OpenGWAS database (https://opengwas.io/) (Table 1). Hypertension outcomes were sourced from 2 large, independent European cohorts: UK Biobank (Neale Lab release, n = 462,933; GWAS ID: ukb-b-7582) and the FinnGen R8 release (n = 218,754; GWAS ID: finn-b-I9_HYPTENS). All original studies obtained ethical approval from their respective institutional review boards and secured informed consent from participants; therefore, this secondary analysis required no additional ethics oversight.^[33–35]^

**Table 1:** Summary of GWAS information.

Exposure/outcome	Year	Population	Consortium	Simple size	Number of SNPs	PMID	GWAS ID
BFP	2021	European	NA	401,772	10,783,701	34594039	ebi-a-GCST90013975
Hypertension	2018	European	Neale Lab	462,933	9,851,867	NA	ukb-b-7582
Hypertension	2021	European	NA	218,754	16,380,466	NA	finn-b-I9_HYPTENS

BFP = body fat percentage, GWAS = genome-wide association study, SNP(s) = single-nucleotide polymorphism(s).

#### 2.3.2. Instrument selection and validity

**Figure 3.**
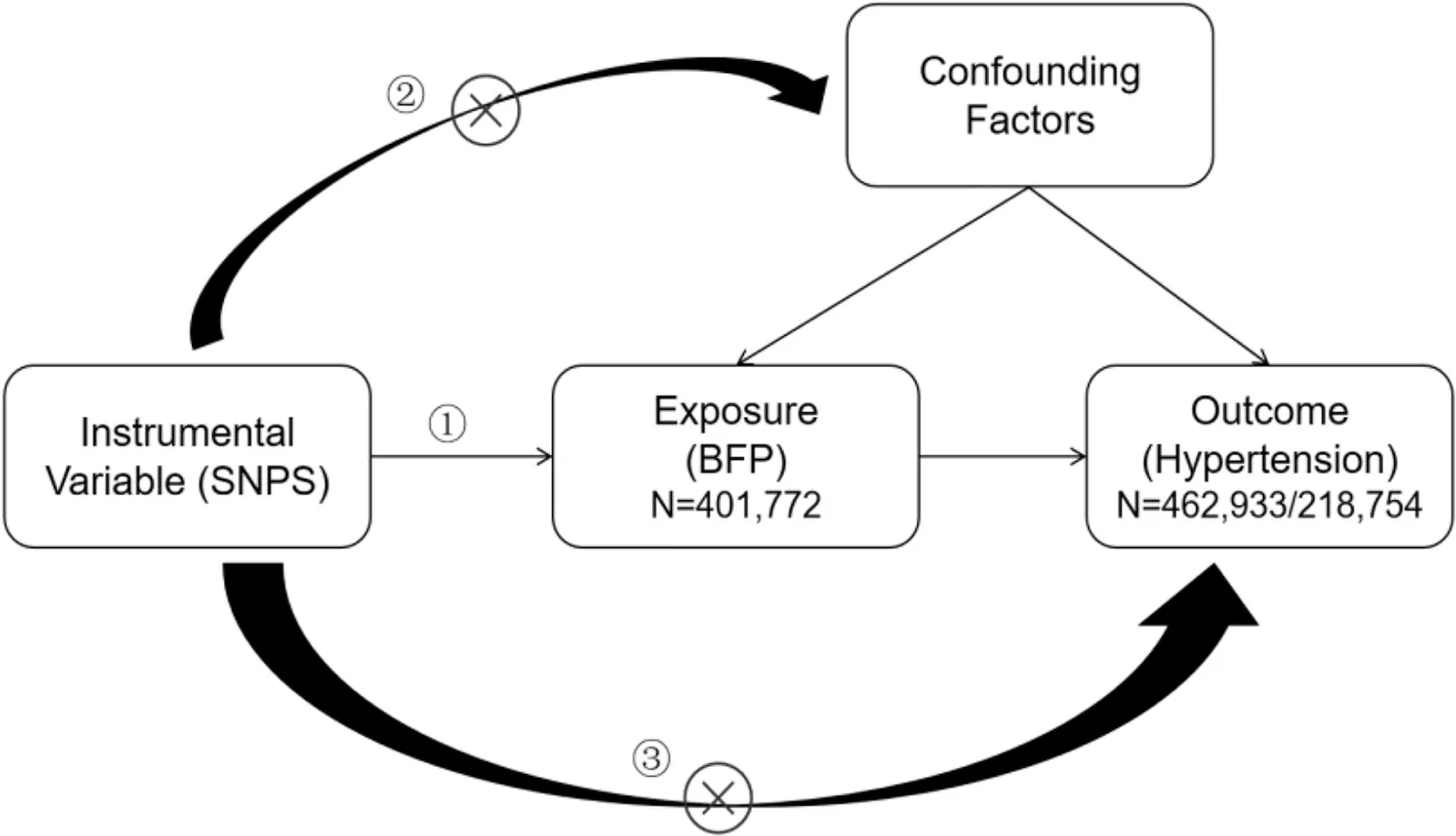
MR analysis flow chart. MR = Mendelian randomization.

MR relies on 3 core assumptions for valid causal inference: relevance (genetic variants are strongly associated with the exposure); independence (variants are unconfounded); and exclusion restriction (variants affect the outcome only through the exposure) (Fig. 3). To satisfy these criteria, we implemented a stringent single-nucleotide polymorphism (SNP) selection protocol: discovery threshold: we selected independent SNPs associated with BFP at genome-wide significance (*P* < 5 × 10^−8^); linkage disequilibrium pruning: to ensure independence, SNPs in linkage disequilibrium (*r*^2^ ≥ 0.001 within a 10,000-kb window, based on the 1000 Genomes European reference panel) were clumped, retaining only the most significant variant per locus; harmonization: alleles were aligned between exposure and outcome datasets; palindromic SNPs with intermediate allele frequencies (0.43 < minor allele frequency < 0.57) were excluded to avoid strand ambiguity; instrument strength: the *F*-statistic for each SNP was calculated as *F* = β^2^/SE^2^, where *β* and SE denote the SNP–BFP effect estimate and its standard error. All retained instruments exceeded the conventional threshold of *F* > 10, minimizing bias from weak instrument bias.^[36–38]^

The final instrument comprised 187 independent SNPs for BFP (see Table S1, Supplemental Digital Content, https://links.lww.com/MD/R623 for full list and *F*-statistics).

#### 2.3.3. Statistical analysis

We employed the inverse-variance weighted (IVW) method as the primary estimator of causal effect, which provides the most efficient and asymptotically unbiased estimate under the assumption of balanced pleiotropy or its absence.^[20,22,25]^ To assess robustness, we complemented IVW with MR-Egger regression and the weighted median estimator (methods resilient to invalid instruments under distinct assumptions).^[39]^

Sensitivity analyses were conducted to evaluate key MR validity conditions: heterogeneity among SNP-specific causal estimates was assessed using Cochran *Q* statistic; *P* > .05 indicated absence of significant heterogeneity; horizontal pleiotropy was evaluated via the MR-Egger intercept test (*P* > .05 suggests no directional pleiotropy); influential SNP analysis was performed using a leave-one-out permutation to confirm that the overall estimate was not driven by any single variant; visual diagnostics included funnel plots and forest plots.^[40]^ MR effect estimates were interpreted on the scale of the exposure GWAS, reflecting the unit or standardized increase in genetically predicted BFP as defined in each source dataset.

All analyses were implemented in R version 4.2.1 using the TwoSampleMR and MR-PRESSO packages, following best-practice guidelines for MR reporting.^[41]^

## 3. Results

### 3.1. Observational study

#### 3.1.1. Participant characteristics

From the 1999 to 2018 NHANES cycles (n = 101,316), we identified 25,957 adults with complete data on BFP, hypertension status, and covariates (Fig. 2). The prevalence of hypertension in this nationally representative sample was 29.9% (n = 7762).

Compared with normotensive individuals, those with hypertension were significantly older, more likely to be non-Hispanic Black, divorced or widowed, obese (BMI ≥ 30), current/former smokers, and to report diabetes, cardiovascular disease, or cancer (all *P* < .001; Table 2). Notably, poverty-income ratio did not differ between groups (*P* = .689), suggesting that socioeconomic stratification alone cannot account for the observed disparities.

**Table 2:** Baseline characteristics of participants by hypertension status (NHANES 1999–2018).

Participant characteristics	Participant			*P*-value
	Health (N = 18,195)	Hypertension (N = 7762)	Total (N = 25,957)	
Age (yr), median (IQR)	39.0 (29.0, 52.0)	59.0 (46.0, 69.0)	45.0 (32.0, 60.0)	<.001
Gender, n (%)				.005
Male	8654 (47.6%)	3544 (45.7%)	12,198 (47%)	
Female	9541 (52.4%)	4218 (54.3%)	13,759 (53%)	
Race/ethnicity, n (%)				<.001
Mexican American	3305 (18.2%)	959 (12.4%)	4264 (16.4%)	
Other Hispanic	1452 (8%)	537 (6.9%)	1989 (7.7%)	
Non-Hispanic White	8858 (48.7%)	3932 (50.7%)	12,790 (49.3%)	
Non-Hispanic Black	2994 (16.5%)	1844 (23.8%)	4838 (18.6%)	
Other race	1586 (8.7%)	490 (6.3%)	2076 (8%)	
BMXBMI (kg/m^2^), n (%)				<.001
<25	6599 (36.3%)	1418 (18.3%)	8017 (30.9%)	
25–30	6194 (34%)	2590 (33.4%)	8784 (33.8%)	
≥30	5402 (29.7%)	3754 (48.4%)	9156 (35.3%)	
BFP, n (%)				<.001
Adipopenia	2185 (12%)	151 (1.9%)	2336 (9%)	
Normal	9252 (50.8%)	2498 (32.2%)	11,750 (45.3%)	
Obesity	6758 (37.1%)	5113 (65.9%)	11,871 (45.7%)	
Education, n (%)				<.001
Below high school	3610 (19.9%)	1626 (20.9%)	5236 (20.1%)	
High school graduate	3986 (21.9%)	1012 (13%)	5868 (22.6%)	
Above high school	10,599 (58.2%)	4254 (54.8%)	14,853(57.2%)	
PIR, n (%)				.689
<1.3	4731 (26%)	1983 (25.5%)	6714 (25.9%)	
1.3–3.5	6707 (36.9%)	2897 (37.3%)	9604 (37%)	
>3.5	6757 (37.1%)	2882 (37.1%)	9639 (37.1%)	
Marital, n (%)				<.001
Married/partner	11,194 (61.5%)	4808 (61.9%)	16,002 (61.6%)	
Widowed	584 (3.2%)	787 (10.1%)	1371 (5.3%)	
Divorced/separated	2234 (12.3%)	1364 (17.6%)	3598 (13.9%)	
Never married	4183 (23%)	803 (10.3%)	4986 (19.2%)	
Smoking status				<.001
Never smokers	9541 (52.4%)	3396 (43.8%)	12,937 (49.8%)	
Previous smokers	3988 (21.9%)	2631 (33.9%)	6619 (25.5%)	
Current smokers	4666 (25.6%)	1735 (22.4%)	6401 (24.7%)	
Drinking status, n (%)				<.001
No drink	5895 (32.4%)	3231 (41.6%)	9126 (35.2%)	
Moderate alcohol consumption	10,082 (55.4%)	3822 (49.2%)	13,904 (53.6%)	
Excessive alcohol consumption	2218 (12.2%)	709 (9.1%)	2927 (11.3%)	
Diabetes,n (%)				<.001
No	17,413 (95.7%)	6267 (80.7%)	23,680 (91.2%)	
Yes	782 (4.3%)	1495 (19.3%)	2277 (8.8%)	
Asthma, n (%)				<.001
No	15,809 (86.9%)	6539 (84.2%)	22,348 (86.1%)	
Yes	2386 (13.1%)	1223 (15.8%)	3609 (13.9%)	
Heart failure, n (%)				<.001
No	18,061 (99.3%)	7358 (94.8%)	25,419 (97.9%)	
Yes	134 (0.7%)	404 (5.2%)	538 (2.1%)	
Coronary heart disease, n (%)				<.001
No	17,954 (98.7%)	7160 (92.2%)	25,114 (96.8%)	
Yes	241 (1.3%)	602 (7.8%)	843 (3.2%)	
Chronic bronchitis, n (%)				<.001
No	17,407 (95.7%)	7152 (92.1%)	24,559 (94.6%)	
Yes	788 (4.3%)	610 (7.9%)	1398 (5.4%)	
Stroke, n (%)				<.001
No	18,023 (99.1%)	7332 (94.5%)	25,355 (97.7%)	
Yes	172 (0.9%)	430 (5.5%)	602 (2.3%)	
Cancer, n (%)				<.001
No	17,162 (94.3%)	6662 (85.8%)	23,824 (91.8%)	
Yes	1033 (5.7%)	1100 (14.2%)	2133 (8.2%)	
Laboratory features				
WBC (U/L)	6.9 (5.7, 8.4)	7.1 (5.8, 8.4)	6.9 (5.7, 8.4)	<.001
RBC (U/L)	4.7 (4.4, 5.1)	4.7 (4.3, 5.0)	4.7 (4.4, 5.0)	<.001
NE (%)	58.3 (52.1, 64.3)	59.2 (52.5, 65.1)	58.7 (52.2, 64.5)	<.001
LY (%)	30.2 (25.0, 35.7)	29.0 (23.5, 34.9)	29.8 (24.6, 35.6)	<.001
PLT (×10^9^/L)	246.0 (211.0, 289.0)	242.0 (203.0, 287.0)	245.0 (208.0, 288.0)	<.001
ALB (g/L)	4.3 (4.1, 4.5)	4.2 (4.0, 4.4)	4.3 (4.1, 4.5)	<.001
SCR (μmol/L)	0.8 (0.7, 1.0)	0.9 (0.8, 1.1)	0.8 (0.7, 1.0)	<.001
GLU (mmol/L)	5.0 (4.6, 5.4)	5.3 (4.9, 6.1)	5.1 (4.7, 5.6)	<.001
TG (mmol/L)	110.0 (74.0, 169.0)	133.0 (90.0, 199.0)	116.0 (78.0, 178.0)	<.001
NA (mmol/L)	139.0 (138.0, 141.0)	139.0 (138.0, 141.0)	139.0 (138.0, 141.0)	<.001
K (mmol/L)	4.0 (3.8, 4.2)	4.0 (3.8, 4.2)	4.0 (3.8, 4.2)	<.001
BUN (mmol/L)	12.0 (9.0, 15.0)	14.0 (11.0, 18.0)	12.0 (10.0, 16.0)	<.001
CI (mmol/L)	104.0 (102.0, 105.0)	103.0 (101.0, 105.0)	103.3 (102.0, 105.0)	<.001
AST (U/L)	22.0 (19.0, 27.0)	23.0 (20.0, 28.0)	23.0 (19.0, 27.0)	<.001
ALT (U/L)	21.0 (16.0, 29.0)	22.0 (17.0, 30.0)	21.0 (16.0, 29.0)	<.001

ALT = alanine aminotransferase, AST = aspartate aminotransferase, BFP = body fat percentage, BUN = blood urea nitrogen, CI = confidence interval, LY = lymphocyte, NHANES = National Health and Nutrition Examination Survey, PLT = platelet count, RBC = red blood cell count, TG = triglycerides, WBC = white blood cell count.

When stratified by BFP category (adipopenia [men ≤ 17%, women ≤ 30%], normal, and obesity [men > 30%, women > 40%]) participants exhibited marked gradients in demographic, clinical, and metabolic profiles (Table 3). Obesity was strongly associated with older age, male sex, lower educational attainment, higher prevalence of cardiometabolic comorbidities, and adverse laboratory markers (e.g., elevated glucose, triglycerides, creatinine; all *P* < .001). Hypertension prevalence rose steeply across BFP strata: 6.5% in adipopenia, 21.3% in normal, and 43.1% in obesity (a sixfold gradient that persisted across subgroups).

**Table 3:** Baseline characteristics of participants by BFP status (NHANES 1999–2018).

Participant characteristics	Participant				*P*-value
	Adipopenia (N = 2336)	Normal (N = 11,750)	Obesity (N = 11,871)	Total (N = 25957)	
Age (yr), median (IQR)	27.5 (23.0, 35.0)	43.0 (31.0, 57.0)	51.0 (37.0, 64.0)	45.0 (32.0, 60.0)	<.001
Gender, n (%)					<.001
Male	1463 (62.6%)	4513 (38.4%)	6222 (52.4%)	12,198 (47%)	
Female	873 (37.4%)	7237 (61.6%)	5649 (47.6%)	13,759 (53%)	
Race/ethnicity, n (%)					<.001
Mexican American	252 (10.8%)	1917 (16.3%)	2095 (17.6%)	4264 (16.4%)	
Other Hispanic	161 (6.9%)	883 (7.5%)	945 (8%)	1989 (7.7%)	
Non-Hispanic White	1184 (50.7%)	5889 (50.1%)	5717 (48.2%)	12,790 (49.3%)	
Non-Hispanic Black	409 (17.5%)	1891 (16.1%)	2538 (21.4%)	4838 (18.6%)	
Other race	330 (14.1%)	1170 (10%)	576 (4.9%)	2076 (8%)	
BMXBMI (kg/m^2^), n (%)					<.001
<25	2320 (99.3%)	5653 (48.1%)	44 (0.4%)	8017 (30.9%)	
25–30	0 (0%)	5852 (49.8%)	2932 (24.7%)	8784 (33.8%)	
≥30	16 (0.7%)	245 (2.1%)	8895 (74.9%)	9156 (35.3%)	
Education, n (%)					<.001
Below high school	352 (15.1%)	2428 (20.6%)	2456 (20.7%)	5236 (20.1%)	
High school graduate	505 (21.6%)	2496 (21.2%)	2867 (24.2%)	5868 (22.6%)	
Above high school	1479 (63.3%)	6826 (58.1%)	6548 (55.1)	14,853(57.2%)	
PIR, n (%)					<.001
<1.3	730 (31.2%)	2950 (25.1%)	3034 (25.6%)	6714 (25.9%)	
1.3–3.5	842 (36%)	4166 (35.5%)	4596 (38.7%)	9604 (37%)	
>3.5	764 (32.7%)	4634 (39.4%)	4241 (35.7%)	9639 (37.1%)	
Marital, n (%)					<.001
Married/partner	1083 (46.4%)	7485 (63.7%)	7434 (62.6%)	16,002 (61.6%)	
Widowed	19 (0.8%)	461 (3.9%)	891 (7.5%)	1371 (5.3%)	
Divorced/separated	187 (8%)	1574 (13.4%)	1837 (15.5%)	3598 (13.9%)	
Never married	1047 (44.8%)	2230 (19%)	1709 (14.4%)	4986 (19.2%)	
Smoking status					<.001
Never smokers	1263 (54.1%)	5757 (49%)	5917 (49.8%)	12,937 (49.8%)	
Previous smokers	269 (11.5%)	2761 (23.5%)	3589 (30.2%)	6619 (25.5%)	
Current smokers	804 (34.4%)	3232 (27.5%)	2365 (19.9%)	6401 (24.7%)	
Drinking status, n(%)					<.001
No drink	678 (29%)	3888 (33.1%)	4560 (38.4%)	9126 (35.2%)	
Moderate alcohol consumption	1394 (59.7%)	6383 (54.3%)	6127 (51.6%)	13,904 (53.6%)	
Excessive alcohol consumption	264 (11.3%)	1479 (12.6%)	1184 (10%)	2927 (11.3%)	
Diabetes,n (%)					<.001
No	2318 (99.2%)	11,169 (95.1%)	10,193 (85.9%)	23,680 (91.2%)	
Yes	18 (0.8%)	581 (4.9%)	1678 (14.1%)	2277 (8.8%)	
Asthma, n (%)					<.001
No	1986 (85%)	10,290 (87.6%)	10,072 (84.8%)	22,348 (86.1%)	
Yes	350 (15%)	1460 (12.4%)	1799 (15.2%)	3609 (13.9%)	
Heart failure, n (%)					<.001
No	2330 (99.7%)	11,583 (98.6%)	11,506 (96.9%)	25,419 (97.9%)	
Yes	6 (0.3%)	167 (1.4%)	365 (3.1%)	538 (2.1%)	
Coronary heart disease,n (%)					<.001
No	2330 (99.7%)	11,458 (97.5%)	11,326 (95.4%)	25,114 (96.8%)	
Yes	6 (0.3%)	292 (2.5%)	545 (4.6%)	843 (3.2%)	
Chronic bronchitis, n (%)					<.001
No	2235 (95.7%)	11,259 (95.8%)	11,065 (93.2%)	24,559 (94.6%)	
Yes	101 (4.3%)	491 (4.2%)	806 (6.8%)	1398 (5.4%)	
Stroke, n (%)					<.001
No	2319 (99.3%)	11,551 (98.3%)	11,485 (96.7%)	25,355 (97.7%)	
Yes	17 (0.7%)	199 (1.7%)	386 (3.3%)	602 (2.3%)	
Cancer, n (%)					<.001
No	2270 (97.2%)	10,894 (92.7%)	10,660 (89.8%)	23,824 (91.8%)	
Yes	66 (2.8%)	856 (7.3%)	1211 (10.2%)	2133 (8.2%)	
Hypertension, n (%)					<.001
No	2185 (93.5%)	9252 (78.7%)	6758 (56.9%)	18,195 (70.1%)	
Yes	151 (6.5%)	2498 (21.3%)	5113 (43.1%)	7762 (29.9%)	
Laboratory features					
WBC (U/L)	6.6 (5.5, 8.0)	6.7 (5.6, 8.1)	7.3 (6.0, 8.7)	6.9 (5.7, 8.4)	<.001
RBC (U/L)	4.5 (4.3, 4.9)	4.7 (4.4, 5.1)	4.7 (4.4, 5.0)	4.7 (4.4, 5.0)	<.001
NE (%)	57.6 (51.1, 63.9)	58.1 (51.9, 64.3)	59.2 (52.7, 64.8)	58.7 (52.2, 64.5)	<.001
LY (%)	30.8 (25.5, 36.7)	30.1 (24.7, 35.7)	29.5 (24.4, 35.1)	29.8 (24.6, 35.6)	<.001
PLT (×10^9^/L)	242.0 (209.0, 284.0)	242.0 (207.0, 284.0)	249.0 (210.0, 294.0)	245.0 (208.0, 288.0)	<.001
ALB (g/L)	4.4 (4.2, 4.6)	4.4 (4.1, 4.6)	4.2 (4.0, 4.4)	4.3 (4.1, 4.5)	<.001
SCR (μmol/L)	0.8 (0.7, 0.9)	0.9 (0.7, 1.0)	0.8 (0.7, 1.0)	0.8 (0.7, 1.0)	<.001
GLU (mmol/L)	4.7 (4.4, 5.0)	5.0 (4.7, 5.4)	5.3 (4.8, 5.9)	5.1 (4.7, 5.6)	<.001
TG (mmol/L)	76.0 (56.0, 105.0)	107.0 (73.0, 163.0)	137.0 (94.0, 205.0)	116.0 (78.0, 178.0)	<.001
NA (mmol/L)	139.0 (138.0, 140.0)	139.0 (138.0, 141.0)	139.0 (138.0, 141.0)	139.0 (138.0, 141.0)	<.001
K (mmol/L)	3.9 (3.7, 4.1)	4.0 (3.8, 4.2)	4.0 (3.8, 4.2)	4.0 (3.8, 4.2)	<.001
BUN (mmol/L)	11.0 (9.0, 13.0)	12.0 (10.0, 16.0)	13.0 (10.0, 16.0)	12.0 (10.0, 16.0)	<.001
CI (mmol/L)	103.0 (102.0, 105.0)	103.0 (101.8, 105.0)	104.0 (102.0, 105.0)	103.3 (102.0, 105.0)	<.001
AST (U/L)	21.0 (18.0, 25.0)	23.0 (20.0, 28.0)	23.0 (19.0, 28.0)	23.0 (19.0, 27.0)	<.001
ALT (U/L)	16.0 (13.0, 21.0)	21.0 (16.0, 28.0)	23.0 (17.0, 31.0)	21.0 (16.0, 29.0)	<.001
LDH (U/L)	116.0 (103.0, 131.0)	126.0 (112.0, 144.0)	134.0 (118.0, 153.0)	129.0 (113.0, 147.0)	<.001

ALT = alanine aminotransferase, AST = aspartate aminotransferase, BFP = body fat percentage, BUN = blood urea nitrogen, CI = confidence interval, LDH = lactate dehydrogenase, LY = lymphocyte, NHANES = National Health and Nutrition Examination Survey, PLT = platelet count, RBC = red blood cell count, TG = triglycerides, WBC = white blood cell count.

#### 3.1.2. Association between BFP and hypertension

In unadjusted analysis, obesity conferred a 6.88-fold higher odds of hypertension relative to adipopenia (odds ratio [OR] = 6.88, 95% confidence interval [CI]: 5.82–8.13; *P* < .001; Table 4). This association remained robust after progressive adjustment for sociodemographics (Model I), comorbidities (Model II), and laboratory biomarkers (Model III).

**Table 4:** Univariate logistic regression analysis of hypertension risk in NHANES 1999 to 2018.

Characteristics	Participant			OR, 95% CI, *P*-value
	Adipopenia (N = 2336)	Normal (N = 11,750)	Obesity (N = 11,871)	
Hypertension, n (%)				
No	2185 (93.5%)	9252 (78.7%)	6758 (56.9%)	
Yes	151 (6.5%)	2498 (21.3%)	5113 (43.1%)	6.88 (5.82–8.13, *P* < .001)
Gender, n (%)				
Male	1463 (62.6%)	4513 (38.4%)	6222 (52.4%)	
Female	873 (37.4%)	7237 (61.6%)	5649 (47.6%)	2.01 (1.84–2.20, *P* < .001)
Race/ethnicity, n (%)				
Mexican American	252 (10.8%)	1917 (16.3%)	2095 (17.6%)	
Other Hispanic	161 (6.9%)	883 (7.5%)	945 (8%)	0.71 (0.58–0.88, *P* = .001)
Non-Hispanic White	1184 (50.7%)	5889 (50.1%)	5717 (48.2%)	0.62 (0.53–0.71, *P* < .001)
Non-Hispanic Black	409 (17.5%)	1891 (16.1%)	2538 (21.4%)	0.68 (0.58–0.80, *P* < .001)
Other race	330 (14.1%)	1170 (10%)	576 (4.9%)	0.33 (0.28–0.40, *P* < .001)
DMDEDUC2	70 (3%)	919 (7.8%)	912 (7.7%)	
	282 (12.1%)	1509 (12.8%)	1544 (13%)	0.41 (0.32–0.54, *P* < .001)
	505 (21.6%)	2496 (21.2%)	2867 (24.2%)	0.41 (0.31–0.52, *P* < .001)
	774 (33.1%)	3382 (28.8%)	3991 (33.6%)	0.36 (0.28–0.47, *P* < .001)
	705 (30.2%)	3444 (29.3%)	2557 (21.5%)	0.33 (0.25–0.42, *P* < .001)
PIR, n (%)				
<1.3	730 (31.2%)	2950 (25.1%)	3034 (25.6%)	
1.3–3.5	842 (36%)	4166 (35.5%)	4596 (38.7%)	1.27 (1.14–1.41, *P* < .001)
>3.5	764 (32.7%)	4634 (39.4%)	4241 (35.7%)	1.42 (1.27–1.58, *P* < .001)
Marital, n (%)				
Married/partner	1083 (46.4%)	7485 (63.7%)	7434 (62.6%)	
Widowed	19 (0.8%)	461 (3.9%)	891 (7.5%)	5.17 (3.27–8.16, *P* < .001)
Divorced/separated	187 (8%)	1574 (13.4%)	1837 (15.5%)	1.32 (1.13–1.55, *P* < .001)
Never married	1047 (44.8%)	2230 (19%)	1709 (14.4%)	0.27 (0.25–0.30, *P* < .001)
Smoking status				
Never smokers	1263 (54.1%)	5757 (49%)	5917 (49.8%)	
Previous smokers	269 (11.5%)	2761 (23.5%)	3589 (30.2%)	2.55 (2.23–2.92, *P* < .001)
Current smokers	804 (34.4%)	3232 (27.5%)	2365 (19.9%)	0.75 (0.69–0.83, *P* < .001)
Drinking status, n (%)				
No drink	678 (29%)	3888 (33.1%)	4560 (38.4%)	
Moderate alcohol consumption	1394 (59.7%)	6383 (54.3%)	6127 (51.6%)	0.72 (0.65–0.79, *P* < .001)
Excessive alcohol consumption	264 (11.3%)	1479 (12.6%)	1184 (10%)	0.81 (0.70–0.94, *P* = .005)
Diabetes,n (%)				
No	2318 (99.2%)	11,169 (95.1%)	10,193 (85.9%)	
Yes	18 (0.8%)	581 (4.9%)	1678 (14.1%)	13.62 (8.55–21.70, *P* < .001)
Asthma, n (%)				
No	1986 (85%)	10,290 (87.6%)	10,072 (84.8%)	
Yes	350 (15%)	1460 (12.4%)	1799 (15.2%)	0.91 (0.81–1.02, *P* = .114)
Heart failure, n (%)				
No	2330 (99.7%)	11,583 (98.6%)	11,506 (96.9%)	
Yes	6 (0.3%)	167 (1.4%)	365 (3.1%)	8.95 (4.00–20.00, *P* < .001)
Coronary heart disease, n (%)				
No	2330 (99.7%)	11,458 (97.5%)	11,326 (95.4%)	
Yes	6 (0.3%)	292 (2.5%)	545 (4.6%)	14.27 (6.38–31.88, *P* < .001)
Stroke, n (%)				
No	2319 (99.3%)	11,551 (98.3%)	11,485 (96.7%)	
Yes	17 (0.7%)	199 (1.7%)	386 (3.3%)	3.46 (2.13–5.62, *P* < .001)
Chronic bronchitis, n (%)				
No	2235 (95.7%)	11,259 (95.8%)	11,065 (93.2%)	
Yes	101 (4.3%)	491 (4.2%)	806 (6.8%)	1.29 (1.05–1.58, *P* = .017)
Cancer, n (%)				
No	2270 (97.2%)	10,894 (92.7%)	10,660 (89.8%)	
Yes	66 (2.8%)	856 (7.3%)	1211 (10.2%)	3.30 (2.57–4.23, *P* < .001)
Age	30.3 ± 9.6	44.9 ± 16.4	50.9 ± 16.8	1.06 (1.05–1.06, *P* < .001)
WBC	6.9 ± 2.1	7.0 ± 2.5	7.5 ± 2.3	1.10 (1.07–1.12, *P* < .001)
RBC	4.6 ± 0.5	4.7 ± 0.5	4.7 ± 0.5	1.70 (1.56–1.85, *P* < .001)
NE	57.5 ± 9.8	57.9 ± 9.6	58.6 ± 9.3	1.01 (1.00–1.01, *P* < .001)
LY	31.3 ± 8.6	30.5 ± 8.6	30.1 ± 8.4	0.99 (0.98–0.99, *P* < .001)
PLT	249.8 ± 62.9	248.9 ± 63.1	256.3 ± 68.5	1.00 (1.00–1.00, *P* = .050)
ALB	4.4 ± 0.4	4.3 ± 0.3	4.2 ± 0.3	0.22 (0.20–0.26, *P* < .001)
Scr	0.8 ± 0.3	0.9 ± 0.4	0.9 ± 0.3	6.01 (4.84–7.46, *P* < .001)
Glu	4.8 ± 1.0	5.3 ± 1.6	5.8 ± 2.1	2.49 (2.32–2.67, *P* < .001)
TG	88.8 ± 51.5	136.9 ± 110.7	169.4 ± 125.4	1.01 (1.01–1.02, *P* < .001)
K	3.9 ± 0.3	4.0 ± 0.3	4.0 ± 0.3	1.99 (1.75–2.27, *P* < .001)
Na	139.0 ± 2.2	139.2 ± 2.4	139.2 ± 2.4	1.03 (1.01–1.05, *P* = .001)
CI	103.2 ± 2.6	103.2 ± 2.9	103.4 ± 3.0	1.01 (1.00–1.03, *P* = .093)
BUN	11.0 ± 3.9	13.1 ± 4.9	13.7 ± 5.8	1.13 (1.12–1.14, *P* < .001)
AST	23.2 ± 13.7	26.2 ± 24.7	25.7 ± 16.3	1.02 (1.02–1.03, *P* < .001)
ALT	19.1 ± 12.7	25.9 ± 29.3	27.5 ± 20.3	1.06 (1.06–1.07, *P* < .001)
LDH	119.5 ± 26.0	130.4 ± 34.4	137.8 ± 31.5	1.02 (1.02–1.02, *P* < .001)

ALT = alanine aminotransferase, AST = aspartate aminotransferase, BUN = blood urea nitrogen, CI = confidence interval, LDH = lactate dehydrogenase, LY = lymphocyte, NHANES = National Health and Nutrition Examination Survey, OR = odds ratio, PLT = platelet count, RBC = red blood cell count, TG = triglycerides, WBC = white blood cell count.

In the fully adjusted model, individuals with normal BFP had 1.75 times higher odds (95% CI: 1.46–2.10), while those with obesity had 3.80 times higher odds (95% CI: 3.15–4.58) of hypertension compared to the adipopenic reference group (Table 5). A per-unit increase in continuous BFP was also independently associated with higher hypertension risk (OR = 1.07 per 1% increase, 95% CI: 1.06–1.08; *P* < .001).

**Table 5:** Weighted multivariable-adjusted logistic regression of hypertension risk in NHANES 1999 to 2018.

Exposure	Crude model	Model I	Model II	Model III
BFP 3 categorical groups				
Adipopenia	Ref	Ref	Ref	Ref
Normal	3.50 (2.95–4.16, *P* < .001)	1.77 (1.48–2.11, *P* < .001)	1.78 (1.49–2.14, *P* < .001)	1.75 (1.46–2.10, *P* < .001)
Obesity	8.08 (6.71–9.72, *P* < .001)	4.19 (3.51–5.01, *P* < .001)	3.88 (3.25–4.64, *P* < .001)	3.80 (3.15–4.58, *P* < .001)
BFP continuous	1.02 (1.01–1.02, *P* < .001)	1.08 (1.07–1.08, *P* < .001)	1.06 (1.05–1.07, *P* < .001	1.07 (1.06–1.08, *P* < .001

BFP = body fat percentage, NHANES = National Health and Nutrition Examination Survey.

#### 3.1.3. Nonlinear association and sex-specific thresholds

**Figure 4.**
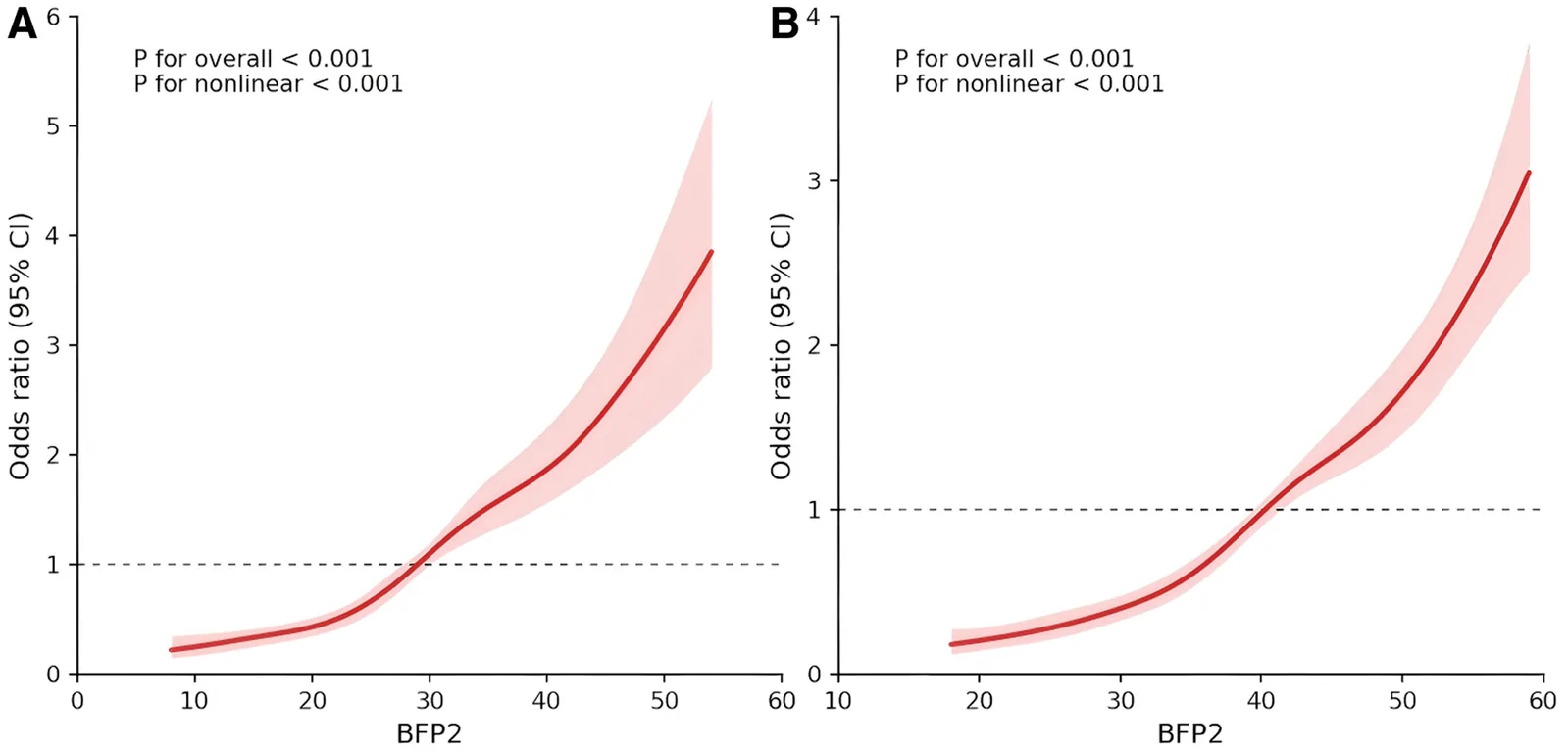
RCS analysis. (A) Males; (B) females. RCS = restricted cubic spline.

Restricted cubic spline modeling confirmed a nonlinear, monotonically increasing relationship between BFP and hypertension in both sexes (nonlinearity *P* < .001; Fig. 4). Segmented regression identified sex-specific inflection points: 31.32% in men and 41.82% in women.

Below these thresholds, each 1% increment in BFP was associated with substantially higher hypertension risk (men: OR = 1.10, 95% CI: 1.08–1.11; women: OR = 1.16, 95% CI: 1.14–1.17; both *P* < .001) (Tables 6 and 7). Above the thresholds, the slope attenuated but remained positive (men: OR = 1.05; women: OR = 1.04), indicating that risk persists but at a diminished marginal rate. These findings underscore a critical window of heightened susceptibility (particularly in men, who reach this inflection at markedly lower BFP levels).

**Table 6:** Threshold effect analysis (male).

Exposure	Adjusted OR (95% CI), *P*-value
BFP	1.07 (1.07–1.08), <.001
Inflection point (K)	31.32
BFP < 31.32	1.10 (1.08–1.11), <.001
BMI ≥ 31.32	1.05 (1.04–1.07), <.001
Log likelihood ratio	.002

BFP = body fat percentage, BMI = body mass index, CI = confidence interval, OR = odds ratio.

**Table 7:** Threshold effect analysis (female).

Exposure	Adjusted OR (95% CI), *P*-value
BFP	1.10 (1.09–1.10), <.001
Inflection point (K)	41.82
BFP < 41.82	1.16 (1.14–1.17), <.001
BMI ≥ 41.82	1.04 (1.03–1.06), <.001
Log likelihood ratio	<.001

BFP = body fat percentage, BMI = body mass index, CI = confidence interval, OR = odds ratio.

### 3.2. Mendelian randomization study

**Figure 5.**
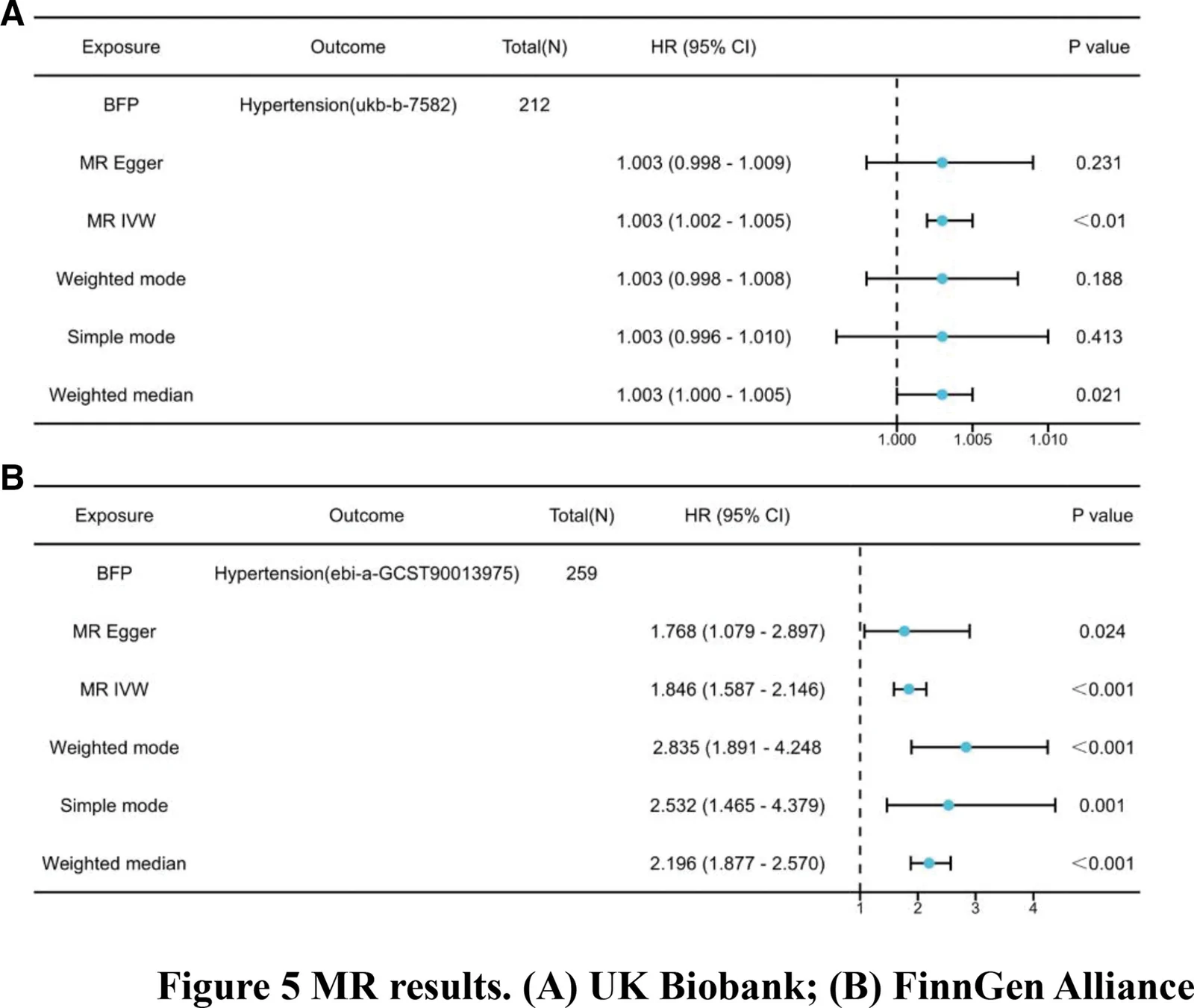
MR results. (A) UK Biobank; (B) FinnGen Alliance. FinnGen = Finnish Genetic Epidemiology Consortium, MR = Mendelian randomization, UK Biobank = United Kingdom Biobank.

Genetically predicted higher BFP was causally associated with increased hypertension risk in both UK Biobank and FinnGen cohorts. In the IVW analysis, each 1% genetically elevated BFP conferred: OR = 1.003 (95% CI: 1.001–1.005; *P* < .001) in UK Biobank (n = 462,933); OR = 1.846 (95% CI: 1.587–2.146; *P* < .001) in FinnGen (n = 218,754; Fig. 5).

All instrumental SNPs satisfied the weak instrument criterion (*F* > 10; Table S1, Supplemental Digital Content, https://links.lww.com/MD/R623). Sensitivity analyses confirmed robustness: no heterogeneity: Cochran *Q P* = .16–0.31 (Table 8); no directional pleiotropy: MR-Egger intercept *P* = .858–0.967 (Table 9); no outlier-driven effects: Leave-one-out and funnel plot analyses showed consistent directional estimates across all SNPs (Figs. S1–S3, Supplemental Digital Content, https://links.lww.com/MD/R624).

**Table 8:** Heterogeneity test.

Exposure	Outcome	Method	Cochran *Q*	*P*
BFP (ebi-a-GCST90013975)	Hypertension (ukb-b-7582)	Inverse variance weighted	229	.16
		MR Egger	210	.18
BFP (ebi-a-GCST90013975)	Hypertension (finn-b-I9_HYPTENS)	Inverse variance weighted	258	.31
		MR Egger	257	.19

BFP = body fat percentage, MR = Mendelian randomization.

**Table 9:** Horizontal pleiotropy test.

Exposure	Outcome	Method	Intercept b	*P*-value
BFP (ebi-a-GCST90013975)	Hypertension (ukb-b-7582)	MR Egger	0.000	.967
BFP (ebi-a-GCST90013975)	Hypertension (finn-b-I9_HYPTENS)	MR Egger	0.003	.858

BFP = body fat percentage, MR = Mendelian randomization.

These results provide convergent genetic evidence that BFP is not merely a correlate but a causal determinant of hypertension risk in individuals of European ancestry. The apparent difference in effect magnitude between UK Biobank and FinnGen likely reflects differences in phenotype definitions and scaling of the exposure and outcome GWAS summary statistics, as well as cohort characteristics. Importantly, the direction of effect was consistent across cohorts and supported by sensitivity analyses.

## 4. Discussion

Our study provides convergent epidemiological and genetic evidence that BFP is not only strongly associated with but causally implicated in the development of hypertension. By integrating a nationally representative observational cohort (NHANES, 1999–2018) with two-sample MR using large-scale European GWAS data, we demonstrate that genetically elevated BFP robustly increases hypertension risk (independent of BMI) and identify sex-specific thresholds beyond which the marginal risk increment attenuates. These findings position BFP as a superior adiposity metric for hypertension risk stratification and underscore the need to move beyond BMI in clinical and public health practice.

### 4.1. BFP as a causal and clinically relevant risk factor

Observational analyses revealed a 6-fold gradient in hypertension prevalence across BFP strata (from 6.5% in adipopenia to 43.1% in obesity) persisting after comprehensive adjustment for demographics, lifestyle, comorbidities, and metabolic biomarkers. Crucially, MR analyses confirmed a causal effect: genetically predicted higher BFP increased hypertension odds by 1.85-fold in FinnGen and conferred a 0.3% per 1% BFP increase in UK Biobank. While the latter effect size appears modest, it reflects the lifelong, incremental exposure inherent to MR and aligns with known polygenic architectures of complex traits. Critically, both datasets showed no heterogeneity or directional pleiotropy, and sensitivity analyses confirmed robustness.

This causal inference resolves a key limitation of prior observational work, which (despite consistent positive associations) remains vulnerable to reverse causation and residual confounding.^[34–38]^ Our dual-evidence approach thus advances the field from correlation to causation, reinforcing that adiposity itself (not merely its correlates) drives blood pressure dysregulation.

### 4.2. Beyond BMI: the case for body composition-based risk assessment

The clinical relevance of BFP lies precisely in its independence from BMI. BMI conflates lean and fat mass, misclassifying up to 30% of individuals with normal BMI but elevated BFP (so-called “normal-weight obesity”) as low-risk.^[34,35,41]^ Our data confirm this: even within the normal BMI range, BFP exhibited strong, nonlinear associations with hypertension. Conversely, some with high BMI but low BFP (e.g., athletes) may be falsely labeled high-risk. BFP, by directly quantifying adipose burden, overcomes these misclassifications and offers superior risk discrimination.

This has profound implications for screening. Current hypertension prevention guidelines rely heavily on BMI thresholds. Our findings argue for integrating BFP assessment (via bioelectrical impedance or dual-energy X-ray absorptiometry) into routine health evaluations, particularly for individuals with normal BMI but high cardiometabolic risk.

### 4.3. Sex-specific thresholds: physiology meets precision prevention

These sex-specific inflection points should be interpreted as population-level slope-change estimates derived from cross-sectional modeling rather than diagnostic cutoffs. They may help generate hypotheses and inform risk communication, but require confirmation in prospective cohorts and diverse populations. One of our most salient findings is the sex-dimorphic inflection point: 31.32% in men versus 41.82% in women. Below these thresholds, each 1% BFP increase conferred substantially higher hypertension risk (men: OR = 1.10; women: OR = 1.16); above them, the slope attenuated but remained positive. This suggests a window of heightened vulnerability during early adiposity accumulation.

Biologically, this dimorphism reflects fundamental differences in fat distribution and hormonal milieu. Men preferentially store fat viscerally (metabolically active depots that drive insulin resistance, RAAS activation, and inflammation).^[42]^ Women, by contrast, store more subcutaneous fat, and estrogen exerts cardioprotective effects via nitric oxide-mediated vasodilation and anti-inflammatory actions.^[39]^ Only when adiposity exceeds a higher threshold (overwhelming these protective mechanisms) does hypertension risk accelerate in women. Clinically, these findings suggest that hypertension risk may accelerate at different BFP ranges in men and women. However, any BFP-based targets should be considered exploratory until validated prospectively.

### 4.4. Mechanistic insights: from adipose biology to blood pressure

The causal link between BFP and hypertension is biologically plausible through multiple, interrelated pathways. Adipose tissue functions as an active endocrine organ, and its expansion triggers: chronic inflammation: excess fat mass releases proinflammatory cytokines (interleukin-6, tumor necrosis factor-alpha, C-reactive protein) and reduces anti-inflammatory adiponectin; insulin resistance: driving sodium retention and activating the RAAS; sympathetic overactivity: increasing cardiac output and peripheral resistance; dysregulated adipokine secretion: altered leptin/adiponectin ratios directly impair endothelial function.

Moreover, obesity-predisposing genes (e.g., FTO and MC4R) may influence blood pressure not only through adiposity but also via central nervous system effects on appetite and autonomic tone.^[2,7,31,33]^ Emerging evidence also implicates gut microbiota dysbiosis and epigenetic reprogramming in mediating adiposity–hypertension links, though these pathways require further MR-based validation.

### 4.5. Strengths, limitations, and future directions

Our study’s strengths include the triangulation of observational and genetic evidence, nationally representative sampling, direct BFP measurement, and rigorous MR methodology with extensive sensitivity analyses. The identification of sex-specific thresholds further enhances clinical translatability.

Nonetheless, limitations merit acknowledgments. First, NHANES is cross-sectional, precluding definitive temporal inference (though MR mitigates this concern). Second, while we adjusted for numerous confounders, unmeasured factors (e.g., diet quality, sleep, and psychosocial stress) may persist. Third, MR analyses were restricted to European ancestry, limiting generalizability to other populations. Fourth, BFP was measured via bioelectrical impedance, which (while validated) may be less precise than dual-energy X-ray absorptiometry or MRI. Fifth, our NHANES analyses were based on a complete-case dataset because participants with missing BFP, hypertension status, or covariates were excluded. This may introduce selection bias if missingness is related to adiposity or blood pressure, and the analytic sample may differ from the full NHANES target population.

Future work could evaluate missingness mechanisms and compare key characteristics between included and excluded participants, or apply appropriate multiple-imputation approaches under the complex survey design.

### 4.6. Clinical and public health implications

Our findings argue for a paradigm shift in hypertension prevention. At the individual level, clinicians should consider BFP assessment for patients with normal BMI but other cardiometabolic risk factors. Early intervention (sodium reduction, physical activity, and weight management) may be particularly effective before crossing the sex-specific threshold.

At the population level, public health strategies should move beyond BMI-centric metrics. School- and workplace-based screening could incorporate BFP to identify high-risk youth and adults earlier. Moreover, sex-specific guidelines for healthy body fat ranges may improve risk communication and motivate behavior change.

Finally, in drug development and clinical trials, stratification by BFP and sex could uncover differential treatment effects and optimize therapeutic targeting.

### 4.7. Implications

Our integrative approach (triangulating population-based epidemiology with genetic causal inference) provides compelling evidence that elevated BFP is not merely associated with but causally contributes to hypertension risk, independent of conventional metrics such as BMI. Critically, this relationship is nonlinear and sex-dimorphic, with distinct inflection points at 31.3% in men and 41.8% in women, beyond which the marginal risk increment attenuates. These thresholds likely reflect fundamental differences in fat distribution, hormonal regulation, and metabolic resilience between sexes.

These findings carry immediate clinical and public health relevance. First, they challenge the hegemony of BMI in risk stratification and advocate for routine incorporation of BFP assessment (via bioelectrical impedance or other accessible modalities) into preventive care pathways. Second, they support the development of sex-specific adiposity targets for hypertension prevention, moving precision medicine beyond genetics alone to include body composition phenotypes. Finally, they underscore that early intervention during the pre-threshold phase of adiposity accumulation may yield the greatest cardiovascular benefit.

Future efforts should focus on validating these thresholds in diverse populations, embedding BFP into risk prediction algorithms, and testing whether BFP-guided lifestyle or pharmacologic interventions reduce incident hypertension more effectively than BMI-based strategies. In doing so, we may finally shift the paradigm from weight-centric to adiposity-centric prevention of one of the world’s leading causes of premature death.

## Acknowledgments

Thank you for the relevant funds of Tianjin University of Traditional Chinese Medicine for supporting the publication of this paper.

## Author contributions

**Conceptualization:** Yinuo Qu, Mengyang Wang.

**Data curation:** Yinuo Qu, Chunqi Jiang, Muning Wang, Yirui Chen, Mengyang Wang.

**Formal analysis:** Muning Wang.

**Funding acquisition:** Chunqi Jiang, Yirui Chen.

**Investigation:** Chunqi Jiang.

**Methodology:** Yirui Chen.

**Project administration:** Muning Wang.

**Supervision:** Muning Wang.

**Visualization:** Yinuo Qu.

**Writing – original draft:** Yinuo Qu, Zhiyuan Liu, Ziyi Zhang, Hongyu Li, Hongxin Gui, Zixin Zhang, Mengyang Wang.

**Writing – review & editing:** Mengyang Wang.

## Supplementary Material




