# Supplementary material for: Body fat percentage and hypertension risk: A population-based observational and Mendelian randomization study
Source: Medicine (Baltimore). 2026 Apr 10;105(15):e48262. doi: 10.1097/MD.0000000000048262 (PMC13593337; doi:10.1097/MD.0000000000048262)
Supplement: Supplementary file 1 [file medi-105-e48262-s002.pdf]

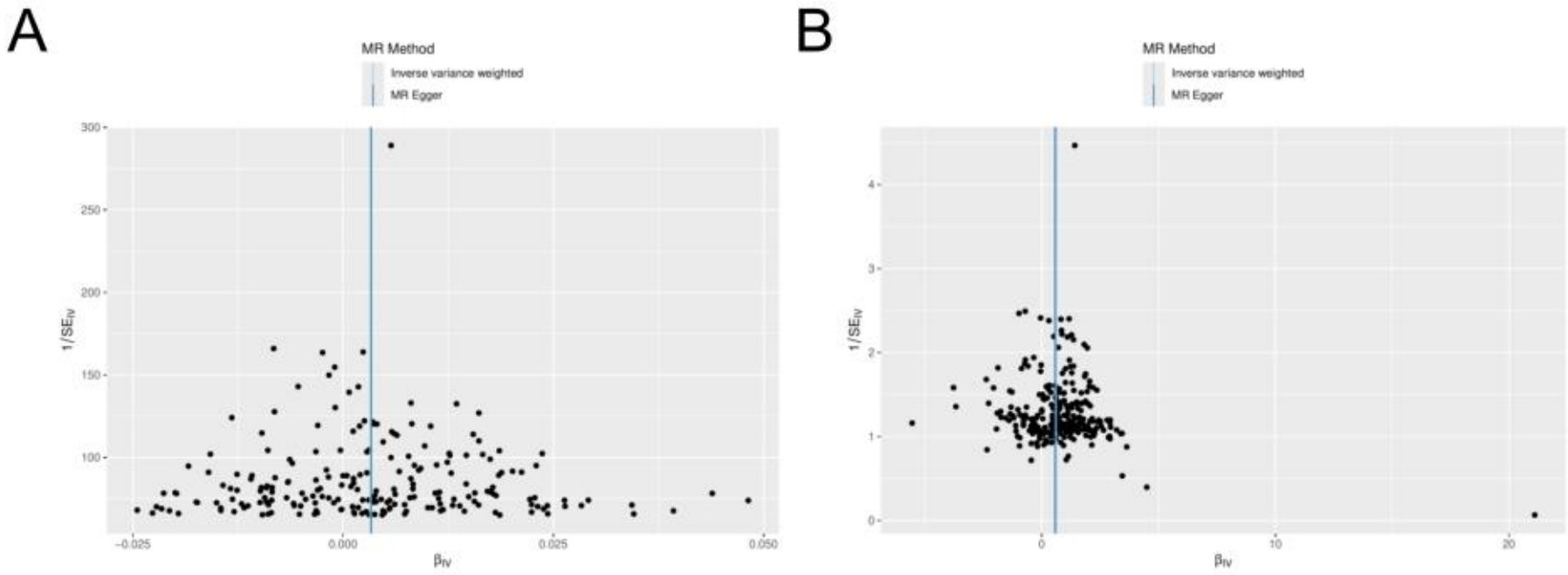

Figure 1 Funnel plot (A)UK Biobank dataset. (B)Finn dataset.

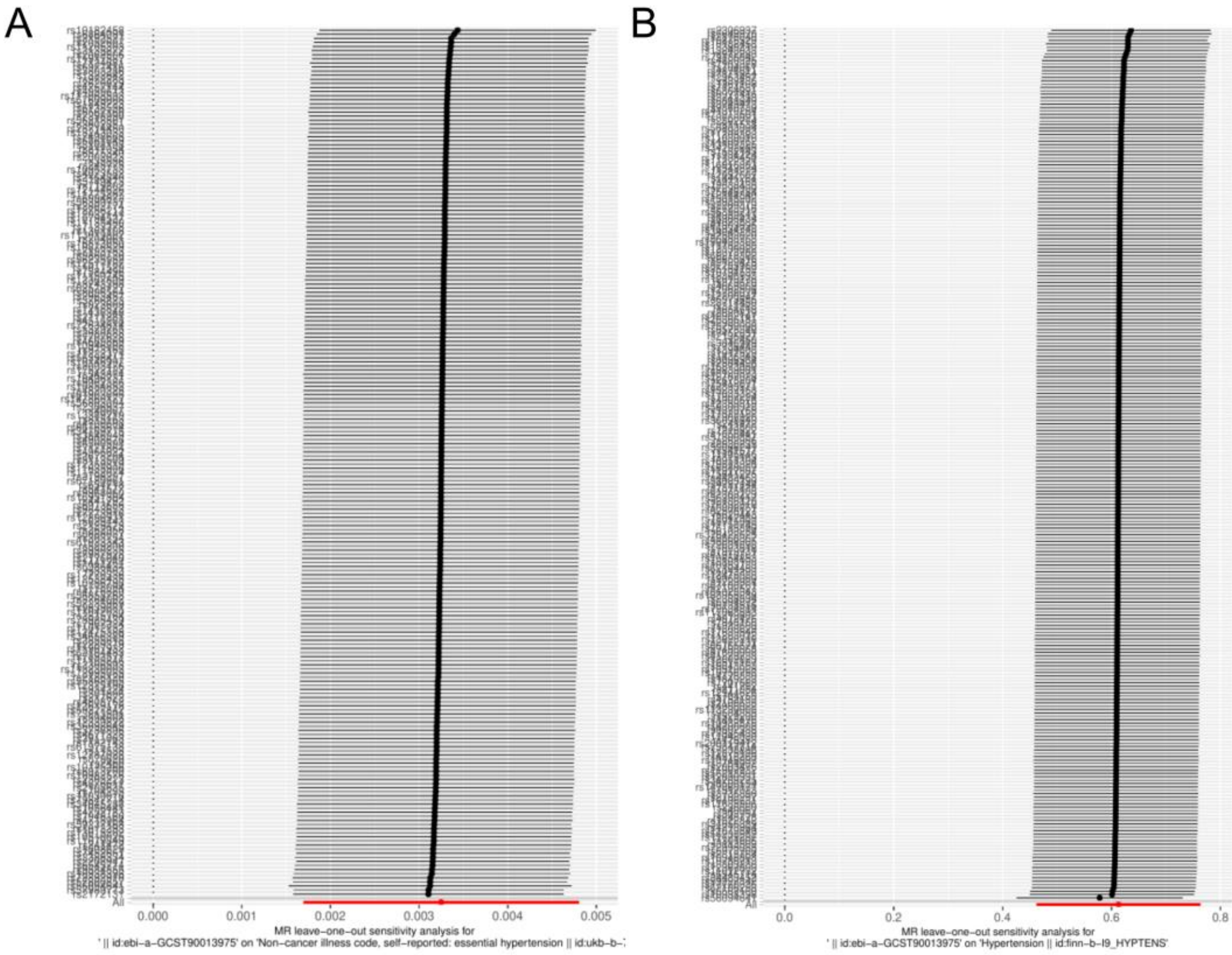

Figure 2 Leave-one-out plot (A)UK Biobank dataset. (B)Finn dataset.

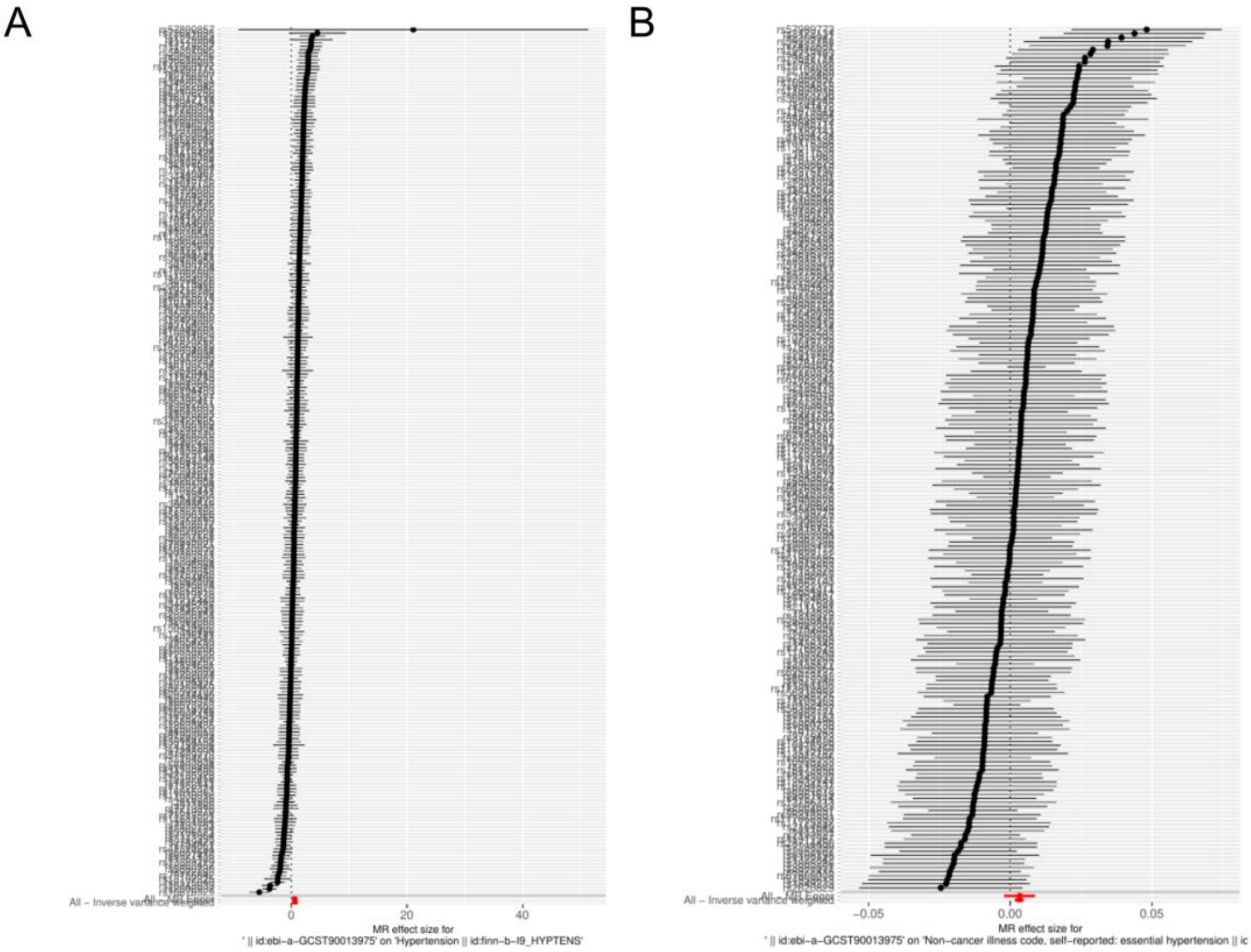

Figure 3 Forest plot (A)UK Biobank dataset. (B)Finn dataset.
